# Barriers to and Facilitators of the Consumption of Animal-Based Protein-Rich Foods in Older Adults

**DOI:** 10.3390/nu8040187

**Published:** 2016-03-29

**Authors:** K. M. Appleton

**Affiliations:** Research Centre for Behaviour Change, Department of Psychology, Bournemouth University, Poole House, Fern Barrow, Poole BH12 5BB, UK; k.appleton@bournemouth.ac.uk; Tel.: +44-1202-965985; Fax: +44-1202-965314

**Keywords:** protein, animal-based foods, food intake, questionnaire, older adults

## Abstract

Protein intakes in the older population can be lower than recommended for good health, and while reasons for low protein intakes can be provided, little work has attempted to investigate these reasons in relation to actual intakes, and so identify those of likely greatest impact when designing interventions. Questionnaires assessing: usual consumption of meat, fish, eggs and dairy products; agreement/disagreement with reasons for the consumption/non-consumption of these foods; and several demographic and lifestyle characteristics; were sent to 1000 UK community-dwelling adults aged 65 years and over. In total, 351 returned questionnaires, representative of the UK older population for gender and age, were suitable for analysis. Different factors were important for consumption of the four food groups, but similarities were also found. These similarities likely reflect issues of particular concern to both the consumption of animal-based protein-rich foods and the consumption of these foods by older adults. Taken together, these findings suggest intakes to be explained by, and thus that strategies for increasing consumption should focus on: increasing liking/tastiness; improving convenience and the effort required for food preparation and consumption; minimizing spoilage and wastage; and improving perceptions of affordability or value for money; freshness; and the healthiness of protein-rich foods.

## 1. Introduction

Ageing is associated with a progressive loss of protein status [1,2,3], resulting in an increased risk of falls and fractures, decreased immune function, increased risk of infection, increased hospital stays, decreased mobility, decreased independence and increased morbidity and mortality [1,2,3,4].

This loss of protein status results from increased requirements, as a result of age-related increases in muscle and bone degradation and increased illness and injury [1,3,5], and from inadequate intakes. Protein intakes impact directly on protein synthesis and turnover [6,7,8,9,10,11,12], and epidemiological studies demonstrate negative associations between protein intakes and the incidence of falls, frailty, osteoporotic fractures, muscle mass and muscle size, bone mineral density, functional abilities and longevity [4,13,14,15,16,17,18,19,20]. Protein supplementation studies also find improvements in muscle mass, bone health and functionality, improved body weight and reduced medical complications following supplementation compared to usual practice [1,21,22,23,24,25].

Based on the evidence, several studies and select committees currently suggest an increased recommended protein intake from 0.8 g/kg/day to 1.0–1.2 g/kg/day for physically healthy older individuals to prevent protein losses [2,3,5,6,7,8,13,18,21,26,27]. Many older individuals across Europe and the US, however, are not consuming even the lower levels of suggested intakes [28,29,30,31,32]. In the UK and US, approximately 10%–30% of community-dwelling older adults consume less protein than recommended [29,30,31,32]; in the Netherlands, 10%–35% of older adults consume less than the recommended levels of protein [27]; and in Nordic countries, 78%–88% of studied participants were estimated to consume less than the recommended intakes of protein [28].

Protein supplementation studies are typically run using oral nutritional supplements (ONS), but ONS can be expensive, and are often unacceptable to most more able adults [33,34]. Fortified or enriched foods can even be unacceptable to many older consumers [35,36]. The British Association for Parenteral and Enteral Nutrition (BAPEN) recommends a “food first” approach [29], and for most more able adults, increased intakes of protein-rich foods may offer a means through which protein losses can be prevented [29]. Animal-based protein-rich foods may be particularly beneficial. Animal-based protein-rich foods typically contribute a high proportion of protein to an older adults’ diet [27,30,37], can provide a higher quality of protein [5,30,37], and associations between a higher consumption of animal-based protein in older individuals and various positive health outcomes, as above, have been reported [1,15,17,19,20,26].

Food intakes, including intakes of protein-rich foods, however, are known to decline with age [1,3,5,21,27,28,29,30,31,32]. Declines in intakes are largely attributed to deteriorations in appetite, changes in chemosensory abilities, and deteriorations in dentition, manual dexterity, and gastro-intestinal function [38,39,40,41,42,43,44,45,46], and if older individuals themselves are asked, multiple reasons for reduced food consumption can be provided [47,48]. Specific to the consumption of protein-rich foods, we recently found reasons for reduced consumption from older individuals to center around reductions in chemosensory, dental and physical abilities, and changes in living situation [49].

However, while the elucidation of reasons using qualitative methods is of value, little work currently aims to investigate these reasons in relation to actual intakes. By understanding the reasons most related to low protein intakes in a large population-based sample, interventions can target those reasons of greatest potential impact on intakes. Interventions, thus, will have greater impact, and will target those in greatest need, and those of likely greatest benefit. This study aimed to investigate the barriers to and facilitators of intakes of animal-based protein-rich foods in relation to actual intakes, in a large sample of community-dwelling older adults. The work was undertaken with a view to recommending future interventions, or areas for intervention, where these recommendations would be based on an increased understanding of root causes.

## 2. Methods

A questionnaire assessed protein intakes, the barriers and facilitators associated with protein intakes, and various demographic and lifestyle characteristics in an older age group. Associations between intakes and barriers and facilitators of importance were subsequently determined by regression.

### 2.1. Questionnaire

The questionnaire assessed current usual consumption of several animal-based protein-rich foods, barriers and facilitators to the consumption of those foods, and various demographic and lifestyle characteristics of potential impact on consumption, in that order.

### 2.2. Usual Consumption

Usual consumption of the protein-rich foods was assessed using a food frequency questionnaire (FFQ) asking for the frequency of consumption of: white meat (e.g., chicken, turkey); red meat (e.g., beef, lamb, pork); processed meats (e.g., ham, bacon, sausages); white fish (e.g., cod, haddock); oily fish (e.g., sardines, salmon); seafood (e.g., prawns, mussels, crab); eggs; milk; yoghurt, custards and blancmanges; soft cheeses (e.g., cream cheese, Dairylea, camembert); and hard cheeses (e.g., cheddar, stilton, emmental). Response options were: “every day”, “3–5 times a week”, “1–2 times a week”, “1–2 times a fortnight”, “1–2 times a month”, “less than once a month”, and “never”, and were scored: 1, 0.57, 0.21, 0.11, 0.05, 0 and 0, respectively, to provide a frequency relative to once per day. Foods were grouped as “meat”—white meat, red meat and processed meats; “fish”—white fish, oily fish and seafood; “eggs”; and “dairy products”—milk, yoghurt, custards and blancmanges, soft cheeses and hard cheeses; for analyses. Questions on plant-based proteins, through questions on nuts; mushrooms; beans; pulses (e.g., lentils, Dahl) and protein substitutes (e.g., Quorn), were also included in the questionnaire to allow for the inclusion of vegetarian diets, and improve the face validity of the questionnaire. The specific foods included were usual components of the UK diet [31], and the response format was taken from a validated FFQ [50].

### 2.3. Barriers to and Facilitators of Consumption

Barriers and facilitators were assessed by asking individuals to agree or disagree with 38 statements referring to various aspects of animal-based protein-rich foods. These statements were composed following the identification of 19 themes from analyses of the conversations of four focus groups on barriers and facilitators to protein-rich foods in older adults (see Best and Appleton [49] for further details). These themes related to: liking; taste; texture; smell; appearance; freshness; quality; food origins; availability; cost; convenience; effort involved; physical abilities; living alone; spoilage and waste; health beliefs; medical concerns; media reports, and habits. Two statements were provided per theme, e.g., for the theme of availability—“I find it difficult to find meat that I like or want to eat where I usually shop” and “The range of meat where I shop is good” —and for the theme of cost—“I am able to afford to eat meat” and “I find meat expensive”. Statements were responded to on a seven-point scale—“strongly agree”, “moderately agree”, “slightly agree”, “neither agree nor disagree”, “slightly disagree”, “moderately disagree”, “strongly disagree”, scored +3 to −3, such that higher scores denote greater agreement. All statements were provided for the four animal-based protein-rich food groups—meat, fish, eggs and dairy products. Participants were asked to only complete each section if they consumed items from that food group.

### 2.4. Demographic and Lifestyle Characteristics

Demographic characteristics assessed by direct questioning were: gender; age; marital status; living status; area of the country of residence (assessed by postcode); Index of Multiple Deprivation (IMD) for the area of residence (assessed by postcode) [51,52,53,54] (a measure of deprivation for an area based on: income; employment; education, skills and training; health deprivation and disability; crime; barriers to housing and services; and living environment deprivation; used as an approximation for socio-economic status); years of education; and nationality. Lifestyle characteristics assessed were: Body Mass Index (BMI) based on self-reported height and body weight; denture wearing; presence of physical disabilities that hinder food purchasing, food preparation or food consumption; and frequency with which individuals received help with food shopping, received help with food cooking, had food delivered, and ate away from their home. Denture wearing was assessed using response options “none”, “partial”, “full” (scored 0, 0.5, 1). Physical disabilities were assessed using response options “no”, “yes, a few”, “yes, some” and “yes, a lot” (scored 0, 0.33, 0.66, 1, respectively), and frequency with which individuals received help, had food delivered and ate out of the home were assessed and scored using response options as for the intake questions. All demographic and lifestyle variables have previously been associated with consumption in older adults [38,43,44,48,55], and may have implications for possible solutions to low intakes.

### 2.5. Questionnaire Administration

The questionnaire was sent to 1000 individuals aged 65 years and over from across the UK living in their own homes. Based on previous similar work [55], we anticipated sufficient responses from 1000 individuals for our proposed primary analyses. Names and contact details for 1000 individuals representative of the UK population according to the 2011 census were gained from a data sampling company (SampleAnswers, London, UK). Questionnaires were sent by post to all individuals in June 2013, and then again in January 2014, June 2014, and January 2015 to non-responders. The complete questionnaire was piloted prior to use to ensure clarity and took 15–20 min to complete. Ethical approval for the study was gained prior to commencement from the Research Ethics Committee of Bournemouth University, UK.

### 2.6. Analyses

Data were first compared to the UK 2011 census using Chi-Squared tests to assess representativeness, and analysed using descriptive statistics. Secondly, associations between intake frequency for each of the separate foods and the demographic and lifestyle variables were investigated using multiple regression (enter method), where frequency of intake of each food was predicted using all demographic and lifestyle variables, with the exception of marital status and nationality (Model 1). Marital status and living status were highly correlated, and living status was used in preference to marital status, as the variable of greatest likely impact on food consumption (e.g., [56]). Nationality was not found to vary sufficiently in the sample to allow investigation. Food groups were analysed separately (a composite measure of total protein intake was not created), because protein content, portion sizes and factors associated with consumption are likely to differ widely between the protein-rich foods studied. Season of questionnaire completion was also included in these analyses in case differences were found [57]. Thirdly, the importance of each barrier and facilitator associated with consumption was assessed in relation to intake of each of the individual food groups using multiple linear regression (enter method), where each barrier and facilitator was included as a predictor of frequency of intake of each food group (Model 2). “Taste” and “liking”, and “smell” and “living alone” were found to correlate highly for all protein-rich foods prior to these analyses, thus only “liking” and “living alone”, respectively, were used in regression analyses. “Freshness” was also found to correlate highly with “quality” in reference to eggs and dairy products, thus only “quality” was used in these analyses. Finally, the importance of each barrier and facilitator associated with consumption was assessed in relation to intake of each of the individual food groups alongside the demographic and lifestyle characteristics using multiple linear regression (enter method) (Model 3). Missing data for demographic and lifestyle variables were completed with means for all regression analyses to allow contribution from all other variables. Areas of residence were ordered south to north for regression analyses, and Indices of Multiple Deprivation were divided by the total number of scores per country to provide comparable approximate scores from 0 to 1 (median in each country and across the UK = 0.5), where lower scores represent greater deprivation. Findings from all three models may offer solutions for preventing low protein intakes in the older population, but findings from models 2 and 3 on barriers and facilitators will demonstrate factors that may be more amenable to change. Findings from models 2 and 3 are of primary interest for informing interventions, while findings from model 1 are also included for completeness and to offer additional explanation.

## 3. Results

A total of 351 (35.1%) questionnaires were returned and were considered suitable for analysis (251 from summer waves and 100 from winter waves). A further eight questionnaires were returned but were unsuitable for analysis, 25 questionnaires were returned because the addressee was unknown at the address provided, and seven were returned because the addressee had died.

### 3.1. Respondents

Of the 351 respondents, 149 (42%) were male and 202 (58%) were female; 95 (27%) were aged 65–69 years, 90 (26%) were aged 70–74 years, 70 (20%) were aged 75–79 years, 49 (14%) were aged 80–84 years, 17 (5%) were aged 85–89 years, and three (1%) were aged 90–94 years; 209 (60%) were married and 127 (36%) were not/no longer married; 208 (60%) lived with others while 133 (38%) lived alone; and 146 (42%) individuals lived in the South of the UK (South East, South West, London), 89 (25%) lived in the Midlands and Wales (East of England, East Midlands, West Midlands, Wales), 67 (19%) lived in the North of England (North East, North West, Yorkshire), and 45 (13%) lived in Scotland and Northern Ireland. All except 10 individuals reported their nationality as English, Welsh, Scots, Northern Irish or British. Scores for deprivation (IMD) ranged from 0.01 to 0.99, with a mean of 0.60 ± 0.24. The sample had a mean of 13 ± 3 years of education (range 8–24 years), and a mean BMI of 31 ± 5 kg/m^2^ (range 16–52 kg/m^2^).

Respondents were representative of the UK older population in terms of gender (χ^2^ = 0.29, *df* = 1, *p* > 0.05) and age (χ^2^ = 4.9, *df* = 3, *p* > 0.05), but more individuals resided in the South than would be expected based on the UK 2011 census (χ^2^ = 18.1, *df* = 3, *p* < 0.05), and particularly the South West.

A total of 185 (53%) individuals did not wear dentures, 111 (31%) individuals wore partial dentures and 55 (16%) individuals wore full dentures. The majority of people had no disabilities that hindered food purchasing, preparation or consumption (*N* = 287 (82%)), although the full range of disabilities was also reported. The majority of the population also did not receive help with food shopping, cooking or had food delivered (*N* = 243 (69%)), although, again, use of all types of help was reported. The majority of individuals (68%) also ate out at least once a month (126 (32%)) individuals never ate out of their home), and 62 (18%) individuals ate out at least once a week.

Men lived more with others, had more years of education, and ate out of the home more frequently than females (smallest χ^2^ = 4.01, *df* = 1, *p* = 0.04). Age was significantly positively correlated with denture wearing (*r* = 0.19, *p* < 0.01), physical disabilities that hinder food purchasing, preparation or consumption (*r* = 0.21, *p* < 0.01), and frequency with which individuals received help or had food delivered (*r* = 0.18, *p* < 0.01). Denture wearing positively correlated with physical disabilities that hinder food purchasing, preparation or consumption (*r* = 0.14, *p* = 0.01), and frequency with which individuals received help or had food delivered (*r* = 0.12, *p* = 0.02). Physical disabilities that hinder food purchasing, preparation or consumption was highly correlated with frequency with which individuals received help or had food delivered (*r* = 0.47, *p* < 0.01). Individuals who lived alone were of a higher age, received help or had food delivered less frequently and ate out of the home more frequently than those who lived with others (smallest *t* (331) = 2.35, *p* = 0.02). Years of education and score for deprivation (IMD) were positively correlated (*r* = 0.21, *p* < 0.01), and both education (*r* = −0.18, *p* < 0.01) and score for deprivation (IMD) (*r* = −0.11, *p* = 0.04) were negatively correlated with denture wearing. BMI was positively correlated with living further north (*r* = 0.11, *p* = 0.05).

### 3.2. Usual Consumption

All respondents consumed some animal-based protein-rich foods. Dairy products were most frequently consumed, followed by meat, fish and then eggs. Meat consumption was also significantly positively correlated with fish (*r* = 0.16, *p* < 0.01) and egg (*r* = 0.13, *p* = 0.02) consumption.

Results of all regression analyses are given in Table 1, Table 2 and Table 3.

#### 3.2.1. Meat

Of 351 respondents, 340 respondents consumed meat. Meat was consumed a mean of 0.6 ± 0.4 times per day (range 0–3 times).

Considering only the demographic and lifestyle characteristics, the frequency of meat consumption was not significantly predicted by any variable. Considering only the barriers and facilitators, meat consumption was significantly predicted by the regression equation, where more frequent meat consumption was associated with greater agreement that meat is liked or tasty, greater agreement that meat is affordable, and greater agreement that meat is convenient.

Where barriers and facilitators and demographic and lifestyle characteristics were considered together, meat consumption was significantly predicted by the regression equation, where more frequent meat consumption was associated with greater liking for meat, greater agreement that meat is healthy, greater agreement that meat is affordable, and greater agreement that meat is convenient, and a lower age. In secondary analyses, the barriers and facilitators associated with a lower age were lower agreement that the texture of meat makes eating meat difficult (*B* = −0.60, *Beta* = −0.18, *p* = 0.01), greater agreement that meat is convenient (*B* = 0.58, *Beta* = 0.15, *p* = 0.01), and lower agreement that physical disabilities to gain and/or prepare meat hinder eating meat (*B* = −1.03, *Beta* = −0.21, *p* < 0.01).

#### 3.2.2. Fish

Of 351 respondents, 329 respondents consumed fish. Fish was consumed a mean of 0.3 ± 0.3 times per day (range 0–2 times).

Considering only the demographic and lifestyle characteristics, the frequency of fish consumption was not significantly predicted by any of these variables. Considering only the barriers and facilitators, fish consumption was significantly predicted by the regression equation, where more frequent fish consumption was associated with greater liking, greater agreement that fish should be fresh, and greater agreement that preparing and consuming fish is low effort.

Where barriers and facilitators were considered alongside demographic and lifestyle characteristics, fish consumption was significantly predicted by the regression equation, where more frequent fish consumption was associated with greater liking, greater agreement that fish should be fresh, and more years of education. In secondary analyses, the barriers and facilitators associated with more years of education were greater disagreement that fish easily spoils or is wasted (*B* = −0.23, *Beta* = −0.14, *p* = 0.05).

#### 3.2.3. Eggs

Of 351 respondents, 312 respondents consumed eggs. Eggs were consumed a mean of 0.3 ± 0.3 times per day (range 0–1 times).

Considering only the demographic and lifestyle characteristics, egg consumption was significantly predicted by the regression equation, where more frequent egg consumption was associated with a higher BMI and with more frequent eating out of the home. Considering only the barriers and facilitators, egg consumption was significantly predicted by the regression equation, where more frequent egg consumption was associated with greater liking, lower perceptions that eggs easily spoil or are wasted, and greater agreement that eggs are convenient.

Where barriers and facilitators were considered alongside demographic and lifestyle characteristics, egg consumption was significantly predicted by the regression equation, where more frequent egg consumption was associated with greater liking, lower perceptions that eggs easily spoil, and greater agreement that eggs are convenient.

#### 3.2.4. Dairy Products

Of 351 respondents, 347 respondents consumed dairy products. Dairy products were consumed a mean of 1.7 ± 0.7 times per day (range 0–3.6 times).

Considering only the demographic and lifestyle characteristics, dairy consumption was significantly predicted by the regression equation, where more frequent dairy consumption was associated with females, living with others as opposed to living alone, living towards the south, and questionnaire completion in the summer as opposed to winter. Considering only the barriers and facilitators, dairy consumption was significantly predicted by the regression equation, where more frequent consumption was associated with greater liking.

Where barriers and facilitators were considered alongside demographic and lifestyle characteristics, dairy consumption was significantly predicted by the regression equation, where more frequent dairy consumption was associated with greater liking.

## 4. Discussion

The findings indicate, firstly, that intakes of all food groups could be successfully predicted using the barriers and facilitators investigated, both with and without consideration of demographic and lifestyle characteristics. Egg and dairy product consumption were also significantly predicted by the demographic and lifestyle characteristics alone, but other characteristics must also play a role for meat and fish intakes. Secondly, various specific barriers, facilitators, and demographic and lifestyle characteristics were found to impact consumption. These barriers and facilitators, as those that best predict high and low intakes, may be suggested as those most suitable for intervention, to allow the greatest impact in those of greatest need.

### 4.1. Meat

More frequent meat consumption was associated predominantly with a lower age, a greater liking for meat, greater agreement that meat is healthy, greater agreement that meat is affordable, and greater agreement that meat is convenient. A lower age was associated particularly with fewer difficulties with eating meat due to texture, lower perceptions of meat as inconvenient, and lower agreement that physical abilities to gain and/or prepare meat can hinder eating meat.

In relation to age, it is well recognised that lower protein intakes are associated with increasing age [5,27,32,37], and studies looking at specific foods or food groups have demonstrated this effect specifically for meat intakes [37,58]. Decreased intakes with age have been attributed to decreases in oral health and abilities as well as in digestive and gastro-intestinal abilities as individuals age [4,38,41,43,44,45,46]. These associations may be particularly pertinent to certain protein-rich foods, including meat, and our analyses confirm these suggestions. Other studies also demonstrate an association between dental abilities and protein intake [4] and meat intake [41,45]. Our analyses, however, also suggest a role for manual dexterity and physical abilities. Difficulties with eating meat are typically given as a result of the tough and fibrous texture of meat, and these same qualities may affect the strength and manual dexterity required for preparing and cutting meat [45,49]. Various previous work also suggests associations between the diets of older adults and the effort involved in food preparation and consumption, particularly as a result of physical disabilities, including arthritis, poor grip strength, poor muscle strength, and restricted mobility [48,59]. Kelsheimer and Hawkins [59] report considerable difficulty with food preparation as a result of reduced manual dexterity and grip strength in older individuals, and Wylie *et al*. [48] report difficulties in older individuals as a result of physical disabilities that hinder food shopping as well as food preparation. Difficulties with food preparation, as well as consumption, furthermore, may be particularly pertinent to several animal-based protein-rich foods, as many of these foods require preparation prior to consumption. Convenience has also previously been described in older aged groups particularly in relation to efforts to prepare and consume meat, while the time element of convenience has been found to be less important [49].

Of the barriers and facilitators, liking/tastiness, healthiness, cost or affordability and convenience were also important for meat consumption. These four factors have previously been reported as the most important considerations for food consumption in general, in adults [60]. Liking and tastiness are also well-recognised drivers of food intake in older adults [33,47,61], and while taste and odour perception is known to deteriorate with age [39,42,62], studies designed to improve liking or the taste of food items by enhancing taste, e.g., through the use of flavour enhancers such as mono-sodium glutamate (MSG), and by adding already-liked tastes and flavours, e.g., through the use of condiments, show increases in target food item consumption [63,64,65,66,67]. Henry *et al*. [65] demonstrated increased consumption of foods by older adults following the addition of natural food flavours, Schiffman and Warwick [67] demonstrated increased consumption of foods with added flavour enhancers compared to foods without flavour enhancers, and Mathey *et al*. [66] demonstrated increased intake and increased body weight following the addition of flavour enhancers to the daily main cooked meal for 16 weeks compared to a control group who received no flavour enhancement. Liking has also been reported as a good predictor of the consumption of meat [68,69,70], and we have previously demonstrated increased intakes of meat in older adults following the addition of liked sauces and seasonings compared to plain items [63,64].

Healthiness or perceived healthiness is also a well known driver of food intakes (e.g., [60,71]), and positive attitudes toward and increased consumption of meat specifically have previously been associated with perceptions of meat as healthy [58,68,69]. Healthiness or perceived healthiness has also previously been found to be particularly important for older individuals where health may be more fragile and more of a concern [60,71,72]. Others also report the higher consumption of other animal-based protein-rich foods investigated in this study, by those who consider them to be healthy [73,74,75,76,77].

Cost or affordability are also likely to be particularly important in older individuals as finances are often limited [48,73]. Various research suggests that the cost of meat is important in meat consumption [68,69,70], and others also suggest that the cost of meat can be prohibitive and can prevent the consumption of meat by older individuals and individuals of other low-income groups [47,48,49,70].

### 4.2. Fish

More frequent fish consumption was predominantly associated with more years of education, greater liking and greater agreement that fish should be fresh. Perceptions of fish preparation and consumption as being of little effort were also important. More years of education were associated with lower perceptions that fish easily spoils or is wasted.

Associations between education and healthy eating are well known [78]. Associations between education and fish consumption particularly have also previously been reported both independently and as part of a relationship with income and/or socio-economic status, where a higher education is typically associated with a higher income and a higher socio-economic status [75,78,79,80,81]. Not all studies report associations between fish consumption and education or income [82], but reported associations are unlikely to be specific to the older population. Impacts may be exaggerated in the older population, however, as a result of low incomes.

Liking is a well-known predictor of food intake (as above), and has previously been reported as a good predictor of fish and seafood consumption [81].

An impact of freshness, particularly for fish and fish products, has also been previously elucidated [78,83]. For fish, as well as for other animal products, freshness is often associated with quality and food safety [78,84,85], and these associations may be particularly salient for fish (where food poisoning can occur very soon after consumption, compared to that from some other foods [83]), and again may be particularly important for older individuals where food poisoning can result in greater suffering compared to that of younger individuals [83].

Issues of spoilage are likely to be linked to those of freshness. Perceptions that fish does not spoil or is not wasted may imply prompt preparation and consumption by consumers, may imply the purchasing of processed fish or fish products with a long shelf-life, such as smoked fish or canned fish, or may imply the consumption of all parts of the fish that are purchased (*i.e.*, that nothing that is purchased is wasted). Prompt preparation and consumption of fish suggests a degree of planning in fish purchasing, where consumers only purchase fish if they plan to consume it shortly, or it may also be linked to perceptions of fish as convenient or of little effort to prepare. Others also provide reports from consumers of fish as convenient [81]. Use of pre-prepared fish or fish products or easy preparation techniques may also explain associations with effort involved, although increased consumption by those who consider fish to require only little effort may also relate to the ease with which fish can be cooked. Consumption of all purchased parts of the fish suggests that low spoilage or wastage may also be linked to income and affordability or value for money. Regardless of the reason, spoilage and wastage are likely to be a particular concern for protein-rich foods as many of these foods are highly perishable, and they may be a concern particularly for older age groups where spoilage may be less easy to detect due to deteriorations in sight and smell, of greater impact due to impaired infection or immune responses [83], and where reduced abilities make food shopping and cooking more effortful and consequently less frequent.

### 4.3. Eggs

More frequent egg consumption was associated with a greater liking for eggs, greater agreement that eggs do not easily spoil or are wasted, and greater agreement that eggs are convenient. More frequent eating out of the home was also important.

Effects due to liking/tastiness are unsurprising. Egg consumption has previously also been associated with liking [73].

Associations with low spoilage and wastage are also unsurprising. Compared to other protein-rich foods, eggs do not spoil quickly and have a long shelf-life relative to other animal-based protein-rich foods [86], and many of the older people of the current era were brought up in a time when eggs were less likely to be refrigerated, and so may even be inclined to extend the recommended shelf-life for eggs due to refrigeration [87]. Low spoilage and wastage are also likely to be related to both convenience and affordability or value for money. A long shelf-life may allow a limited need for meal planning and infrequent or “bulk” purchasing, resulting in perceptions of convenience [86], and in association, a long shelf-life that results in little wastage will also improve value for money [86]. As with fish, low spoilage and wastage, however, may also relate to the fact that all parts of the egg can be consumed, and thus there is no wastage, and so there is increased value for money. Previous work also suggests egg consumption in older individuals for cost-related reasons [73,87].

Perceptions of eggs as convenient, as food items that are easily prepared and consumed, have also been found elsewhere [86,87], but here may also be associated with dental and physical abilities, to some degree. Eggs are not only easy and fast to prepare and consume for all, but eggs are also soft in texture, and so will be more comfortable to prepare and consume for those with limited dental and physical abilities.

The association between egg consumption and eating out is interesting. This effect may be specific to the UK, where one cheap option for eating out is often the “all-day breakfast”, a meal typically composed of eggs, bacon, sausages, tomatoes, mushrooms, baked beans and toast. Anecdotally, these “all-day breakfast” meals appeal to a lot of older individuals, largely due to value for money [87]. These findings, thus, may be also linked to affordability and value for money.

### 4.4. Dairy Products

More frequent dairy product consumption was predominantly associated with greater liking. Being female, living with others as opposed to alone, living towards the south, and questionnaire completion in the summer as opposed to the winter, however, were also important.

Effects due to liking/tastiness are again unsurprising, and have previously been demonstrated in association with dairy consumption [74,76,77].

Differences between genders and effects due to living with others as opposed to living alone may reflect increased (dairy) drink or dessert consumption in females and in communal situations. A possible impact of dairy drink or dessert consumption deserves further investigation. Hydration can be poor among older individuals [29] and dessert consumption could provide a palatable strategy for increasing protein intakes in this population group. Many studies report difficulties for and poorer diets in older individuals as a result of living alone (e.g., [47,48,49]), and increased socialization during meal times has been demonstrated to have beneficial effects on intakes among older population groups in general [88,89].

Associations between dairy consumption and geographic location are possibly a result of availability, availability over the lifetime resulting in habits and familiarity, as has been suggested for other food products [82], or may reflect an increased likelihood of local food purchasing in older consumers compared to those who are younger. More dairy herds, more yoghurt producers and more cheese producers are found in the south of the UK compared to the north. Greater consumption in summer as opposed to winter is a likely reflection of the traditional British diet, where soups and heavier meat-based meals tend to be consumed in winter and replaced in summer with drinks, including dairy-based drinks, and lighter cheese, egg or cold meat–based alternatives.

### 4.5. Strategies for Improving Consumption

These findings suggest that strategies for increasing consumption should focus on increasing liking/tastiness (meat, fish, eggs, dairy products), improving perceptions of convenience and the effort required for food preparation and consumption (meat, fish, eggs), improving understanding and minimizing perceptions of spoilage and wastage (fish, eggs), improving affordability or perceptions of value for money (meat), improving access to fresh or perceptions of fresh foods (fish), and improving perceptions of the healthiness of protein-rich foods (meat).

Strategies to aid liking and tastiness could focus on the use and promotion of complementary condiments and flavoured items, and the use of recipes. Flavour enhancers such as MSG and the use of flavoured foods, such as sauces and seasonings, have previously been found to have impacts on intakes [63,64,65,66,67], and although concerns have been expressed regarding increased salt intakes as a result of increased MSG intakes, these impacts may be mitigated by the use of foods with high concentrations of naturally-occurring MSG [90]. The promotion of tasty recipes and increased use of taster sessions could also be beneficial.

Strategies to aid convenience and low effort in preparation could focus on the promotion of pre-prepared, pre-cooked or easy to prepare and cook animal-based foods and food products, the promotion of easy-to-prepare recipes, and by association, the benefits and value of eating out. Pre-prepared or pre-cooked products, e.g., canned stews, fish cakes, and fish balls, may aid perceptions of convenience and have also been suggested by others (e.g., [84]), but the weight of canned products and difficulties endured in opening cans can cause their own problems. Strategies to aid easy consumption could also include the use of pre-cut or minced meat/fish, slow-cooked meat/fish or softer products such as meat/fish pates, eggs and some dairy products. Analyses of the UK National Diet and Nutrition Survey suggest difficulties in consuming meat steaks due to loss of teeth, but no effects for sliced meat or cheeses [45]. Recent work by Pennings *et al*. [91] also suggests less time and effort are required to consume minced beef compared to a beef steak by older men. Slow-cooking and various forms of processing can also soften texture, and aid consumption. Kossioni and Bellou [41] report easier consumption as a result of increased preparation, and recommend interventions to teach cooking skills and techniques, where possible. Promoting eating out may also be an option. Eating out, however, can be expensive, and may be difficult for some older individuals due to poor mobility and/or residence in rural locations.

Strategies to aid affordability and low spoilage could focus on the promotion of cheaper but still good sources of protein, such as the cheaper cuts of meat and eggs, practical measures such as freezing foods or bulk-buying and sharing foods, and the use of foods with longer shelf-lives such as eggs and tinned, cured or smoked meat/fish products. Use of recipes, demonstrations, lessons or recommendations on cooking techniques may also be of value for encouraging the use of cheaper protein sources, and also on ensuring consumption of all foods purchased, e.g., through increased consumption of soups and stocks. Increased use of freezers may further enhance perceptions of freshness or an increased use of fresher products. An importance of freshness may counter recommendations for the use of more processed products. Success here may require education on the freshness and quality of animal-based foods prior to processing, or more evidence of food processing at source.

Strategies to improve or increase perceptions of healthiness would require improved education. This education may need to focus on the elements of a food item that make it healthy and/or the health conditions for which certain food items are beneficial, or it may need to focus on targeting misperceptions that certain food items are unhealthy and/or that elements of a food item can make it detrimental to health. The restriction of foods that are considered unhealthy may be particularly pertinent to animal-based protein-rich foods that can be high in saturated fats and cholesterol, but consumer understanding of these issues can also be limited [72,74,76]. Various previous studies demonstrate benefits from educational interventions for older adults, particularly where educational messages are clear, simple, tailored or personalised to specific needs, and where experience, incentives, reinforcement and access to health professionals are also provided [72,92,93].

### 4.6. Comparison with Other Studies

Not all reasons for consumption identified in the focus groups were found to predict intakes, nor were some reasons previously identified by others. The existence of reasons that were not associated with protein intakes in our large sample demonstrates the value of our use of a large sample and the use of quantitative methodologies. Small qualitative studies have provided many reasons for consuming/not consuming foods [47,48,49], but to design interventions for greatest impact, these interventions need to be based on the reasons provided by large numbers and/or those of greatest need. Qualitative studies, for example, are often biased by the use of volunteers with an interest in food [47,49], while these individuals are probably not those in greatest need of intervention. In our findings, for example, medical factors and media reports were not found to impact intakes of any of our protein-rich foods, though these reasons have previously been identified in qualitative studies (e.g., [47,49]). The lack of effects in our analyses may suggest a lack of importance for many—*i.e.*, that these reasons are very individual-specific, or alternatively that these reasons do not actually impact intakes. Various work, for example, suggests that older individuals can prefer to use their own judgments in relation to health matters over those of health professionals or journalists [93]. Scepticism towards the advice of others has previously been attributed to both a belief in one’s own awareness and lifetime experiences, and the existence of advice that is too much, frequently changing, contradictory and/or contradicted by individual experience [93].

Some reasons provided for consumption by other researchers were also not found to be important in our sample. Healthiness, for example, has previously been identified as important for fish [82], egg [73] and dairy [74,75,76,77] consumption, as well as for meat consumption. Medical factors have previously been found to be important for dairy consumption [74,76,77]. Food origins have been found to be important for meat [85] and egg [73] consumption. Many of these other studies, however, have involved wider age ranges and/or have focussed specifically on barriers to consumption, while our study focussed more on identifying the determinants of high consumption, and thus the facilitators of consumption, in only older adults.

A few of our predictors were also important for one food group and not for others. While commonalities are obvious, differences between food groups are also of interest. Perceptions of healthiness, for example, were only important for meat consumption. Perceptions of freshness/quality were important for fish consumption, but were not found for meat, egg or dairy consumption. These differences between foods highlight the value of considering each food group independently, the need for interventions to consider each food group separately, and suggest different interventions will be more beneficial for some food groups than for others.

Many of our demographic and lifestyle variables were also highly correlated, as may be expected [38,43,44,47,48], and some of these were also found to impact intakes. Meat consumption was particularly impacted by age (and its correlates) and fish consumption was particularly impacted by education (and its correlates). These variables may be important for explanations, but are less amenable to intervention. Other demographic and lifestyle predictors largely disappeared once reasons for consumption were also considered (Model 3). This finding suggests that the reasons for consumption are more important than the demographic and lifestyle variables (in the main)—a finding that also argues for interventions that are based more on the reasons.

### 4.7. Strengths and Limitations

Our findings tally well with other studies, but considerably expand on qualitative studies through analysis in relation to protein intakes and consideration of a large representative sample. Other factors of importance in intakes have also been suggested elsewhere (e.g., hospitalization), (e.g., [46]), but these factors were not included in our study because they were not mentioned in our earlier work. These factors may add to the explanatory power of our regression models, but may also offer little additional opportunity for intervention. Our models do offer only limited explanatory power, but our focus was on possible intervention more than explanation. The results of the study are also limited in that all responses were from volunteers and were based on self-report. At a 35% response rate, these volunteers may differ from non-responders, but, based on the high distribution of responses received, we think it is unlikely that responder bias would have systematically affected our measures of either protein intake or the factors associated with protein intake. A measure of portion size was not included in our FFQ, thus we were unable to provide a composite measure of protein intake or comparisons between food groups. Portion sizes were not included to keep our measures simple and easy, and to avoid any false assumption that these contributed to our findings. The inclusion of portion sizes to an FFQ measure has previously been suggested to add little validity to findings, while considerably increasing length and complexity [94,95], and previous work suggests difficulties and inaccuracies in reporting by older individuals when complex measures are used [63]. No assessment was also made of whole diet or protein consumption in relation to body weight. Whole diet was not assessed again to minimize participant burden. Consumption based on body weight was not calculated due to the self-reported nature of body weight and the likely unreliability of this measure in the elderly [66]. Our sample was characterised by a high self-reported BMI. This BMI is likely to be inflated, as a result of natural weight loss, and various reports suggest that poor protein status can still occur in those of a high BMI [2,5], but we accept that our sample may not include those most at risk of low protein status. We also took no account of health conditions and related medications, psychological health or physical activity levels. Various work suggests an impact on intake of physical conditions, e.g., cancers, psychological conditions such as depression, and the medications that may be taken to alleviate these conditions [43,44], but these issues tend to affect those older and more infirmed than the community-dwelling adults of interest in this study [43,44]. Repeated work also demonstrates the added benefit of exercise and, particularly, resistance exercise for the more functional outcomes of a high protein consumption [8,12,21,26]. Importance of time of activity, time of activity in relation to intake and type of activity, however, have also been proposed [8,26], and until effects are confirmed, activity levels were not assessed again to limit participant burden.

The impact of any suggested intervention is difficult to ascertain. Many studies report greater effects of increasing intakes in those of low protein status [14,26], limited effects in those of adequate protein status (e.g., [7,20,96]), and possible negative effects as a result of increasing protein intakes in those with adequate protein status [1,5,96], but maintenance of an adequate protein status in individuals at risk of low protein status will guard against the risks associated with low protein status, with resultant benefits for the individual in the future. The establishment of practices and preferences that maintain adequate protein intakes will also extend preventative effects beyond the time frame of any intervention. Some concerns over high protein intakes have been voiced. These concerns centre around possible impacts on renal activity, bone health, satiety and total energy intakes, saturated fat intakes, and in relation to some cancers [3,21]. Concerns regarding the high consumption of certain animal-based foods have also been voiced, both on health and on environmental grounds [97,98,99,100]. These concerns suggest that increasing protein intakes in all individuals or of all protein-rich foods may not be advisable, and that individual care is also required.

## 5. Conclusions

Various factors were found to impact on the consumption of meat, fish, eggs and dairy products in an older population sample. Different factors were important for the four different animal-based protein-rich food groups, but clear similarities were also found. These similarities are likely to reflect the issues of particular concern to both the consumption of animal-based protein-rich foods and the consumption of these foods by older adults. Taken together, our findings suggest consumption to be explained by, and thus that strategies for increasing consumption should focus on: increasing liking and tastiness; improving convenience and the effort required for food preparation and consumption; improving understanding of and minimizing perceptions of spoilage and wastage; improving affordability or perceptions of value for money; and improving perceptions of the freshness and healthiness of protein-rich foods.

## Figures and Tables

**Table 1:** Outcomes of the regression analyses investigating the impact of demographic and lifestyle characteristics for each food group studied.

	Meat			Fish			Eggs			Dairy		
Regression Equation	*R* = 0.22, *R*^2^ = 0.05, adj. *R*^2^ = 0.02, *F*(12, 350) = 1.48, *p* = 0.12			*R* = 0.19, *R*^2^ = 0.04, adj. *R*^2^ = 0.01, *F*(12, 350) = 1.07, *p* = 0.38			*R* = 0.28, *R*^2^ = 0.08, adj. *R*^2^ = 0.05, *F*(12, 350) = 2.46, *p* < 0.01			*R* = 0.29, *R*^2^ = 0.08, adj. *R*^2^ = 0.05, *F*(12, 350) = 2.53, *p* < 0.01		
	*B*	*Beta*	*p*	*B*	*Beta*	*p*	*B*	*Beta*	*P*	*B*	*Beta*	*p*
Gender (male/female)	−0.008	−0.011	0.85	0.043	0.083	0.14	0.001	0.001	0.98	**0.164**	**0.112**	**0.04**
Age (years)	−0.007	−0.102	0.08	−0.001	−0.025	0.66	−0.004	−0.088	0.12	0.010	0.077	0.18
Living status (alone/with others)	0.069	0.090	0.12	−0.014	−0.028	0.63	0.056	0.106	0.06	**0.175**	**0.117**	**0.04**
Area of residence (South/Midlands and Wales/North of England/Scotland and Northern Ireland)	−0.002	−0.005	0.93	−0.001	−0.004	0.93	0.022	0.090	0.09	**−0.080**	**−0.117**	**0.03**
Multiple Index of Deprivation (0–1)	−0.123	−0.078	0.15	0.022	0.021	0.71	−0.100	−0.091	0.09	0.274	0.089	0.10
Years of education (years)	−0.006	−0.047	0.40	0.010	0.103	0.07	0.004	0.042	0.45	0.013	0.049	0.37
Body Mass Index (kg/m^2^)	−0.002	−0.031	0.57	−0.005	−0.101	0.07	**0.006**	**0.125**	**0.02**	−0.002	−0.016	0.77
Denture wearing (0/0.5/1)	0.033	0.033	0.55	−0.028	−0.040	0.47	0.050	0.071	0.19	−0.168	−0.086	0.12
Physical disabilities (0/0.33/0.66/1)	−0.086	−0.112	0.07	0.001	0.002	0.97	−0.014	−0.027	0.66	−0.100	−0.066	0.28
Receive help/food delivered (0–1)	0.056	0.069	0.28	−0.011	−0.019	0.76	−0.038	−0.066	0.29	0.149	0.094	0.13
Eating out (0–1)	0.272	0.096	0.08	0.044	0.023	0.68	**0.247**	**0.124**	**0.02**	0.200	0.036	0.51
Season of questionnaire completion (summer/winter)	0.001	0.002	0.98	0.032	0.057	0.30	−0.021	−0.036	0.50	**−0.204**	**−0.127**	**0.02**

Significant effects are given in bold (*p* < 0.05).

**Table 2:** Outcomes of the regression analyses investigating the impact of each of the barriers and facilitators for each food group studied.

	Meat			Fish			Eggs			Dairy		
Regression Equation	*R* = 0.38, *R*^2^ = 0.15, adj. *R*^2^ = 0.10, *F*(17, 333) = 3.23, *p* < 0.01			*R* = 0.32, *R*^2^ = 0.10, adj. *R*^2^ = 0.06, *F*(17, 333) = 2.14, *p* = 0.01			*R* = 0.38, *R*^2^ = 0.15, adj. *R*^2^ = 0.10, *F*(16, 333) = 3.36, *p* < 0.01			*R* = 0.31, *R*^2^ = 0.09, adj. *R*^2^ = 0.05, *F*(16, 332) = 2.03, *p* = 0.01		
	*B*	*Beta*	*p*	*B*	*Beta*	*p*	*B*	*Beta*	*p*	*B*	*Beta*	*p*
Liking	**0.047**	**0.142**	**0.04**	**0.047**	**0.167**	**0.02**	**0.040**	**0.165**	**0.02**	**0.218**	**0.225**	**<0.01**
Healthiness	0.034	0.119	0.07	0.030	0.093	0.16	0.011	0.051	0.46	0.010	0.019	0.78
Texture	0.012	0.054	0.38	−0.019	−0.097	0.20	−0.010	−0.044	0.55	0.020	0.033	0.70
Appearance	−0.011	−0.045	0.49	0.006	0.035	0.62	−0.001	−0.006	0.93	−0.031	−0.057	0.50
Affordability	**0.040**	**0.123**	**0.03**	−0.023	−0.102	0.08	0.004	0.018	0.75	0.016	0.023	0.68
Fresh	−0.022	−0.093	0.16	**0.025**	**0.143**	**0.04**	-	-	-	-	-	-
Quality	0.028	0.093	0.18	−0.011	−0.053	0.48	0.002	0.009	0.90	−0.039	−0.062	0.38
Origins	−0.010	−0.048	0.47	−0.009	−0.058	0.42	−0.006	−0.040	0.59	0.008	0.020	0.78
Spoilage	−0.002	−0.009	0.88	−0.011	−0.069	0.29	**−0.036**	**−0.197**	**<0.01**	−0.037	−0.090	0.15
Single	−0.010	−0.049	0.44	0.004	0.024	0.72	−0.001	−0.005	0.95	−0.039	−0.078	0.28
Availability	0.007	0.021	0.73	−0.005	−0.026	0.67	−0.020	−0.098	0.12	0.059	0.102	0.12
Convenient	**0.031**	**0.125**	**0.03**	0.000	0.002	0.97	**0.036**	**0.188**	**<0.01**	0.003	0.006	0.93
Able	−0.001	−0.004	0.94	0.009	0.040	0.53	0.015	0.064	0.27	−0.041	−0.070	0.23
Effort	0.005	0.015	0.79	**0.020**	**0.130**	**0.05**	0.014	0.072	0.29	−0.020	−0.039	0.58
Habit	−0.021	−0.077	0.23	−0.003	−0.014	0.82	−0.018	−0.084	0.18	−0.045	−0.079	0.20
Medical	−0.018	−0.064	0.31	0.015	0.063	0.34	0.014	0.057	0.42	−0.033	−0.061	0.36
Media reports	−0.011	−0.036	0.56	0.010	0.051	0.44	0.019	0.086	0.16	−0.031	−0.050	0.44

Significant effects are given in bold (*p* < 0.05).

**Table 3:** Outcomes of the regression analyses investigating the impact of demographic and lifestyle characteristics and each of the barriers and facilitators for each food group studied.

	Meat			Fish			Eggs			Dairy		
Regression Equation	*R* = 0.46, *R*^2^ = 0.21, adj. *R*^2^ = 0.14, *F*(29, 333) = 2.82, *p* < 0.01			*R* = 0.38, *R*^2^ = 0.14, adj. *R*^2^ = 0.06, *F*(29, 333) = 1.73, *p* = 0.01			*R* = 0.43, *R*^2^ = 0.19, adj. *R*^2^ = 0.11 *F*(28, 333) = 2.54, *p* < 0.01			*R* = 0.39, *R*^2^ = 0.15, adj. *R*^2^ = 0.07, *F*(28, 332) = 1.93, *p* < 0.01		
	*B*	*Beta*	*p*	*B*	*Beta*	*p*	*B*	*Beta*	*p*	*B*	*Beta*	*p*
Gender (male/female)	0.075	0.104	0.08	0.045	0.089	0.15	0.002	0.003	0.96	0.136	0.093	0.12
Age (years)	**−0.008**	**−0.126**	**0.03**	−0.000	−0.007	0.90	−0.002	−0.046	0.42	0.008	0.063	0.30
Living status (alone/with others)	0.074	0.100	0.09	−0.013	−0.025	0.69	0.043	0.080	0.16	0.151	0.101	0.10
Area of residence (South/Midlands and Wales/North of England/Scotland and Northern Ireland)	0.009	0.027	0.62	0.003	0.013	0.82	0.021	0.086	0.12	−0.068	−0.100	0.07
Multiple Index of Deprivation (0–1)	−0.125	−0.082	0.13	0.005	0.005	0.93	−0.071	−0.066	0.23	0.182	0.059	0.29
Years of education (years)	−0.009	−0.064	0.25	**0.013**	**0.141**	**0.01**	0.003	0.029	0.60	0.018	0.067	0.25
Body Mass Index (kg/m^2^)	−0.006	−0.080	0.14	−0.005	−0.093	0.10	0.004	0.081	0.14	−0.002	−0.016	0.78
Denture wearing (0/0.5/1)	0.019	0.020	0.71	−0.027	−0.039	0.49	0.062	0.088	0.11	−0.184	−0.092	0.11
Physical disabilities (0/0.33/0.66/1)	−0.094	−0.123	0.10	0.020	0.037	0.59	0.047	0.083	0.22	−0.025	−0.017	0.81
Receive help/food delivered (0–1)	0.068	0.087	0.17	0.011	0.019	0.77	−0.051	−0.088	0.16	0.125	0.080	0.22
Eating out (0–1)	0.185	0.068	0.22	−0.056	−0.029	0.61	0.139	0.071	0.20	0.042	0.008	0.89
Season of completion (summer/winter)	−0.015	−0.019	0.72	0.017	0.031	0.59	−0.011	−0.019	0.72	−0.170	−0.105	0.06
Liking	**0.050**	**0.153**	**0.03**	**0.048**	**0.169**	**0.02**	**0.040**	**0.165**	**0.03**	**0.176**	**0.182**	**0.01**
Healthiness	**0.038**	**0.135**	**0.04**	0.030	0.090	0.18	0.011	0.049	0.47	0.027	0.048	0.48
Texture	0.005	0.024	0.70	−0.018	−0.093	0.23	−0.004	−0.020	0.79	0.016	0.026	0.76
Appearance	−0.010	−0.039	0.54	0.011	0.063	0.39	−0.005	−0.026	0.72	−0.032	−0.059	0.48
Affordability	**0.041**	**0.128**	**0.03**	−0.022	−0.098	0.10	0.005	0.021	0.70	0.018	0.027	0.63
Fresh	−0.026	−0.107	0.11	**0.026**	**0.147**	**0.04**	-	-	-	-	-	-
Quality	0.023	0.077	0.28	−0.011	−0.051	0.50	0.001	0.004	0.95	−0.034	−0.054	0.45
Origins	−0.013	−0.061	0.36	−0.011	−0.072	0.33	−0.004	−0.027	0.72	−0.002	−0.006	0.94
Spoilage	−0.007	−0.029	0.66	−0.009	−0.056	0.41	**−0.035**	**−0.195**	**<0.01**	−0.026	−0.064	0.31
Single	−0.015	−0.071	0.28	0.005	0.034	0.63	0.002	0.009	0.89	−0.057	−0.114	0.12
Availability	0.002	0.008	0.90	−0.006	−0.033	0.59	−0.017	−0.080	0.20	0.055	0.095	0.14
Convenient	**0.037**	**0.153**	**0.01**	0.004	0.022	0.70	**0.026**	**0.140**	**0.04**	0.016	0.029	0.65
Able	−0.028	−0.092	0.21	0.013	0.058	0.45	0.015	0.064	0.34	−0.016	−0.027	0.71
Effort	0.006	0.019	0.74	0.019	0.123	0.08	0.016	0.081	0.25	−0.033	−0.065	0.37
Habit	−0.019	−0.088	0.27	−0.006	−0.031	0.62	−0.015	−0.072	0.26	−0.049	−0.086	0.16
Medical	−0.018	−0.063	0.31	0.014	0.057	0.40	0.015	0.058	0.43	−0.015	−0.028	0.68
Media reports	−0.012	−0.040	0.53	0.010	0.049	0.47	0.019	0.089	0.15	−0.031	−0.049	0.45

Significant effects are given in bold (*p* < 0.05).
